# Mammal recovery inside and outside terrestrial protected areas

**DOI:** 10.1007/s13280-024-02014-7

**Published:** 2024-04-10

**Authors:** Katherine M. Magoulick, Vanessa Hull, Jianguo Liu

**Affiliations:** 1 Department of Integrative Biology, University of California, Berkeley, Berkeley, CA USA; 2 Department of Wildlife Ecology and Conservation, University of Florida, Gainesville, FL USA; 3 Center for Systems Integration and Sustainability, Department of Fisheries and Wildlife, Michigan State University, East Lansing, MI USA

**Keywords:** Biomes, IUCN red list, Mammal populations, Remoteness, Terrestrial protected areas

## Abstract

Protected areas are a key component of global conservation, and the world is aiming to increase protected areas to cover 30% of land and water through the 30 × 30 Initiative under the Post-2020 Global Biodiversity Framework. However, factors affecting their success or failure in regard to promoting mammal population recovery are not well studied, particularly using quantitative approaches comparing across diverse taxa, biomes, and countries. To better understand how protected areas contribute to mammalian recovery, we conducted an analysis of 2706 mammal populations both inside and outside of protected areas worldwide. We calculated the annual percent change of mammal populations within and outside of terrestrial protected areas and examined the relationship between the percent change and a suite of human and natural characteristics including biome, region, International Union for Conservation of Nature (IUCN) protected area category, IUCN Red List classification, and taxonomic order. Our results show that overall mammal populations inside and outside of protected areas are relatively stable. It appears that Threatened mammals are doing better inside of protected areas than outside, whereas the opposite is true for species of least concern and Near Threatened species. We also found significant population increases in protected areas classified as category III and significant population decreases in protected and unprotected areas throughout Oceania. Our results demonstrate that terrestrial protected areas can be an important approach for mammalian recovery and conservation.

**Supplementary Information:**

The online version contains supplementary material available at 10.1007/s13280-024-02014-7.

## Introduction

Human impacts have led to an increasing number of negative outcomes on animal populations, such as habitat destruction and extinction (Sanderson et al. 2002). Currently, there are over 5500 identified mammalian species, and more than 1/5th of them are classified as threatened (i.e., Vulnerable, Endangered, and Critically Endangered) or extinct (IUCN 2022). Between 1996 and 2008 only 24 species of mammals improved their IUCN Red List rank and approximately 7 times that number worsened in rank (Hoffmann et al. 2011). It is imperative to identify species that are in greatest need for conservation (Abbitt and Scott 2001), but also to design conservation strategies that are successful at reversing their decline.

Within terrestrial systems, protected areas (PAs) are one of the most commonly utilized conservation strategies. The Convention on Biological Diversity recognizes protected areas as playing a key role in biodiversity conservation and seeks to expand the terrestrial protected area network (Secretariat of the Convention on Biological Diversity 2022). As part of the Strategic Plan for Biodiversity, the Aichi Biodiversity Target 11 said that by 2020, 17% terrestrial and 10% of marine areas would be protected (Secretariat of the Convention on Biological Diversity 2010). However, this target was not met, and as of 2020, terrestrial protected areas covered over 16.64% of the globe’s terrestrial surface (UNEP-WCMC and IUCN 2020), but the coverage is not equally proportional across countries (Barr et al. 2011). Subsequently, under the Post-2020 Global Biodiversity Framework also known as the Kunming-Montreal Global Biodiversity Framework, governments agreed to the 30 × 30 target—an initiative to cover 30% of the Earth’s land and water in protected areas by 2030 (Secretariat of the Convention on Biological Diversity 2022).

Mammalian ranges are shrinking faster than PAs are expanding (Pacifici et al. 2020) and there is increasing debate as to whether or not protected areas are indeed effective at maintaining populations and bolstering biodiversity (Liu et al. 2001; Leverington et al. 2010; Laurance et al. 2012; Geldmann et al. 2013, 2018; Coetzee et al. 2014; Amano et al. 2018; Williams et al. 2022). It is well established that not all protected areas offer the same level of protection (Dudley 2008). There are many ways of quantifying PA effectiveness, and thus it can be difficult to assess (Rodrigues and Cazalis 2020). Maxwell et al. (2020) identified three ways that PA effectiveness is often measured: adequacy of management resources, reduction of threats to biodiversity, and comparing area-based conservation to no intervention. Our study focuses on the latter by comparing populations in protected and unprotected areas.

In an effort to clarify the differences between protected areas, the IUCN has established Management Categories, also known as Protected Area Categories, which range from I (strict protection) to VI (sustainable use) and indicate the different management objectives of different protected areas (Dudley 2008). The numbering system is also designed to reflect the level of naturalness with I being the most natural and VI being the least (Dudley 2008). Many studies have attempted to determine if there is a difference in effectiveness between more strictly and less strictly protected areas, often with differing results (Joppa and Pfaff 2011; Coetzee et al. 2014; Elleason et al. 2021). The IUCN has also established the IUCN Red List of Threatened Species (Red List) to assess the conservation status and extinction risk of individual species. The Red List uses categories that range from Least Concern to Extinct with increasing extinction risk. As the IUCN itself notes, Red List categories alone should not determine conservation action but can be valuable indicators when combined with spatial and temporal data (Hoffmann et al. 2008).

Many studies have focused on biodiversity loss (Mora and Sale 2011; Venter et al. 2014) and population decline (Geldmann et al. 2013, 2018) in protected areas globally, but to our knowledge this is the first global analysis comparing annual population change of mammals between protected and unprotected areas. Gray et al. (2016) concluded that species richness and abundance are demonstrably higher inside protected areas than outside; however, the same trend may or may not hold true in regard to animal population changes. Within Africa, there is evidence that many animal populations are in decline outside of protected areas as well as inside them (Western et al. 2009; Ogutu et al. 2016), but other regions are understudied with regard to this question.

We conducted a meta-analysis of mammalian population trends inside and outside of terrestrial protected areas by using data from studies which assessed population trends of specific mammal populations or groups of populations. Our objective was to identify characteristics that are correlated to mammal population changes globally and to see if those characteristics differ inside and outside of protected areas. We anticipated that populations in PAs would show greater population growth than those that are outside PAs. In past studies, which included other taxonomic groups, protected area age and size were correlated with population increase (Barnes et al. 2016a, b); likewise, greater protected area remoteness has been shown to be correlated with lower threats to biodiversity (Schulze et al. 2018). We expected these factors would remain important to conservation success when analyzing at a global scale. We predicted that protected areas that were larger and more remote would exhibit greater mammalian population increases.

## Materials and methods

**Fig. 1:**
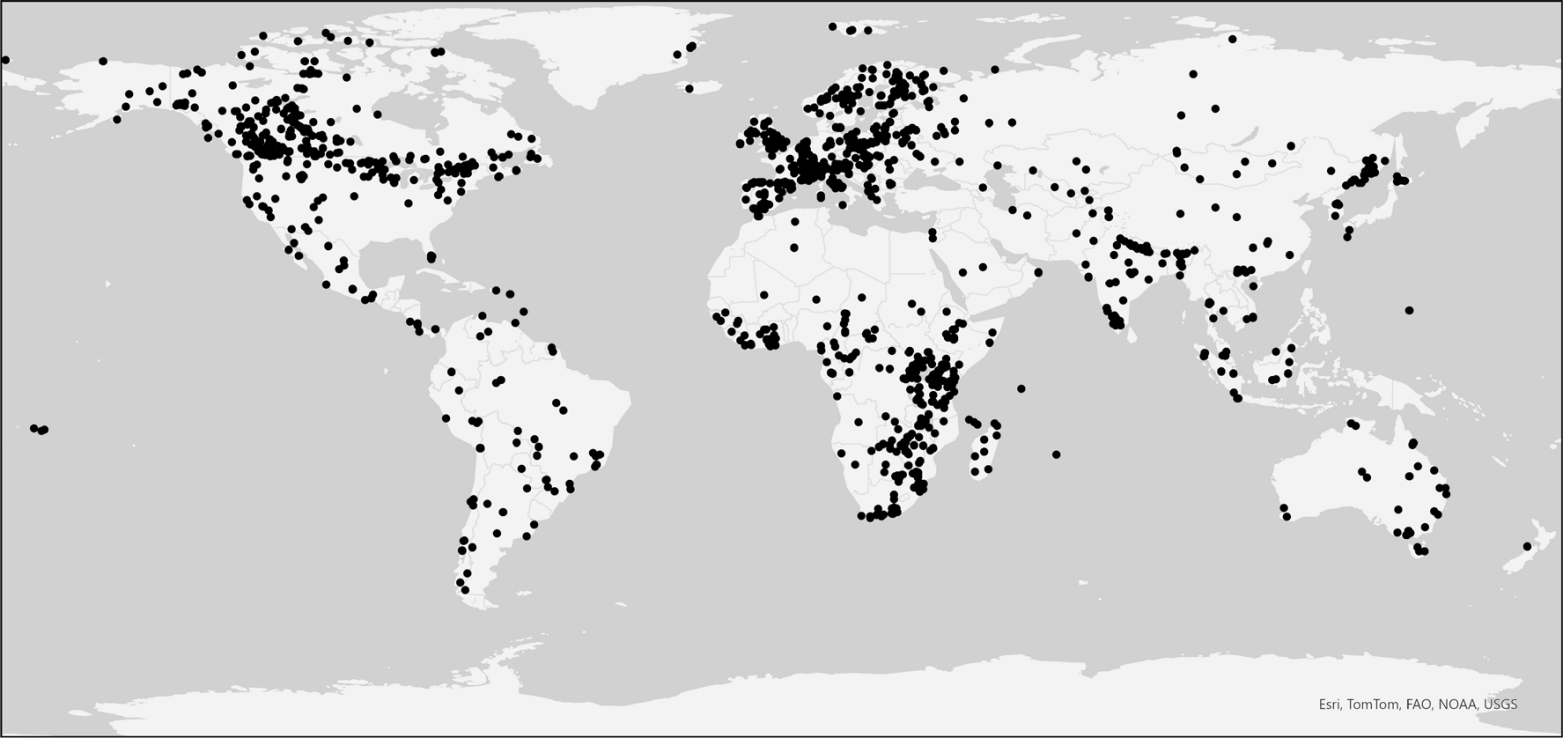
Points indicate the locations of populations sampled in the study

Data on mammalian population trends were downloaded from the Living Planet Database (LPD, accessed 5/18/2022). We removed any data for freshwater or marine species and excluded any populations that were categorized as invasive, as well as any species that are domesticated. We analyzed 2706 mammal populations (Fig. 1), of which 1115 were within 370 different protected areas, 1486 were not protected, and 105 populations spanned both protected and unprotected areas. The populations represent 21 mammalian orders (Table 1), and data points were collected from 1970 to 2014. LPD data is assigned a region: Africa, Asia, Europe, Latin America and the Caribbean, North America, and Oceania. Oceania is defined as the pacific islands including Australia, Melanesia, Micronesia, New Zealand, and Polynesia.

For each species, we retrieved its current IUCN Red List classification (accessed 6/1/2022). Red List categories Vulnerable, Endangered, and Critically Endangered were combined to form one category, “Threatened.” We then manually identified the populations that were located inside PAs by examining the “Location of population”, and for those areas we collected their ID, area (km^2^), IUCN Management Category, governance type, and status year from the World Database on Protected Areas (WDPA) (accessed 3/27/2023). We defined a population as located inside a PA if the original study indicated that the population was located in an area that was designated as protected and matched overall criteria laid out for the definition of protection provided by the WDPA or was in an area which had any of the following key words: park, sanctuary, reserve, conservation area. As mammal populations are found in all six IUCN Management categories, we included populations from all of them. Our search of the WDPA did not explicitly include “other effective area-based conservation measures” (OECMs), but some of the areas that were not found in the WDPA and were manually classified as protected may fit within the definition of OECM. If any data were missing from the WDPA, NA was entered for that category. In some cases, species were not available in the Red List or protected areas were not present in the WDPA and NA was entered accordingly. Protected areas in Asia are underrepresented in the WDPA (You et al. 2018), so these populations, and others with no WDPA or IUCN Red List data were still included in our study, with NAs listed wherever data were missing. Raw data can be found in Table S1.

After separating the data based on whether the location of population was protected, making a note of populations that spanned both protected and non-protected areas, we calculated the annual percent change of each population by subtracting the final population size from the initial population size, dividing by the number of years elapsed, and multiplying by 100. In cases where there were multiple LPD entries from the same species in the same location, we took the average of the annual percent changes. All GIS analyzes were performed in ArcGIS Pro v. 2.9.1 (ESRI Inc 2021).

We calculated the annual percent change for each population and averaged those data for populations of the same species in the same area. We used Wilcoxon signed-rank tests to examine whether mammal annual percent change differed from 0 by mammal order, IUCN Red List category, IUCN protected area category, biome, and region. If two categories in a group (i.e., two different biomes) both showed significant percent changes then we examined group differences with a Wilcoxon test. We used a linear mixed effects model in the R package lme4 v. 1.1.32 (Bates et al. 2015) to relate percent change in population size per year to protected area: area and remoteness with region and order as random effects. Linear mixed effect models allow for the control of random effects, or categorical variables which may be generating noise in the data. All analyses used *α* = 0.05 and were run in R 4.1.3 (R Core Team 2022). We used the LPD coordinates to find the underlying raster attributes that determined remoteness; we used travel time to nearest city as a proxy for remoteness (Weiss et al. 2018).

In this paper when we refer to populations as being protected or unprotected, we are referring to the protection status of the area in which they reside. For example, some populations may not be located in a protected area, but the species are still protected under the law. For the purposes of this study they were still classified as unprotected (i.e., Bobek et al. 2005). 

## Results

**Fig. 2:**
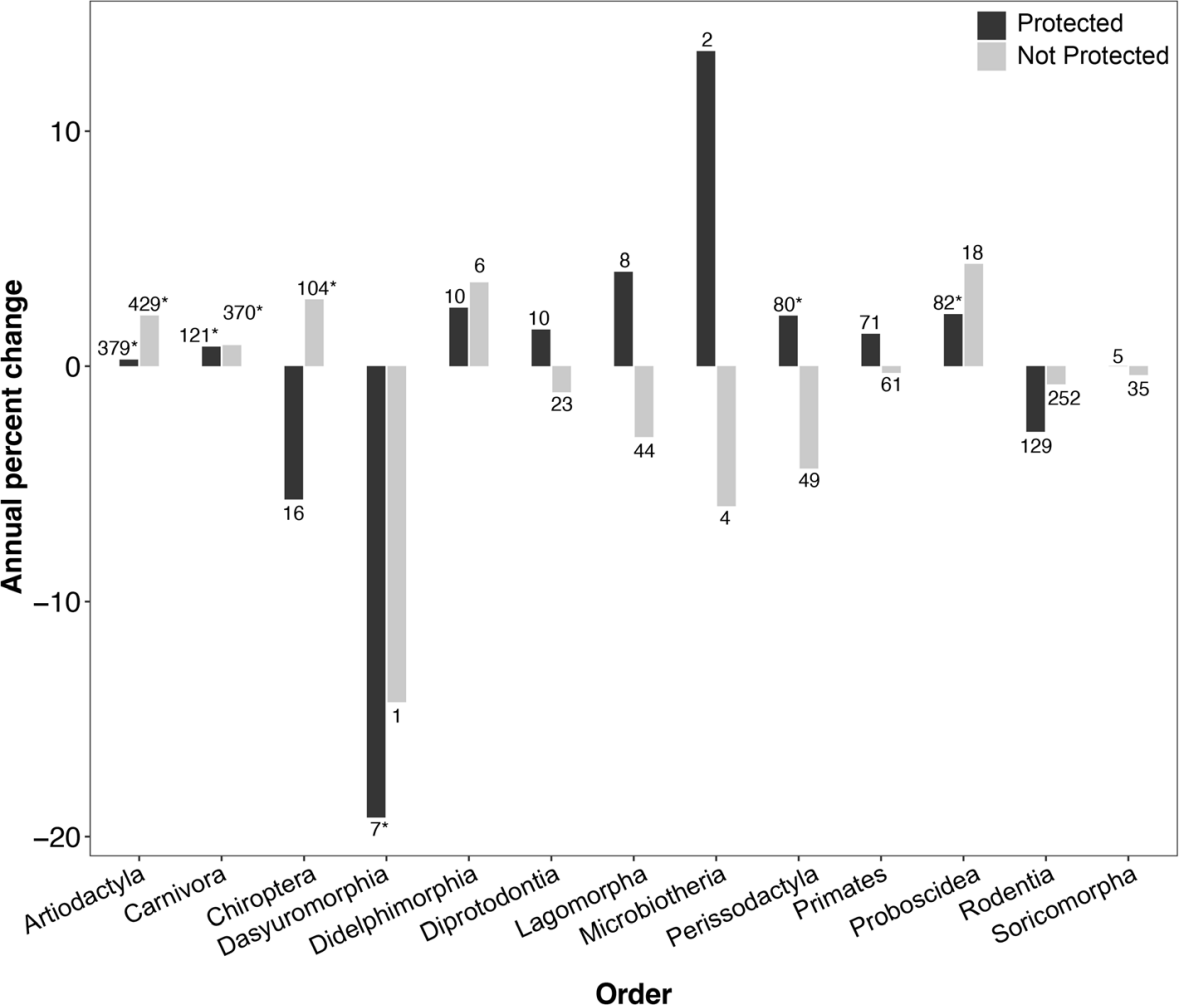
Median mammalian population percent change/year of mammalian orders. The numbers above bars indicate the sample size of populations for each category. Asterisks indicate values that are significantly different from zero. Soricomorpha was used by the Living Planet Database at the time of download, but formally the order has been combined with Erinaceidae to form the order Eulipotyphla

**Fig. 3:**
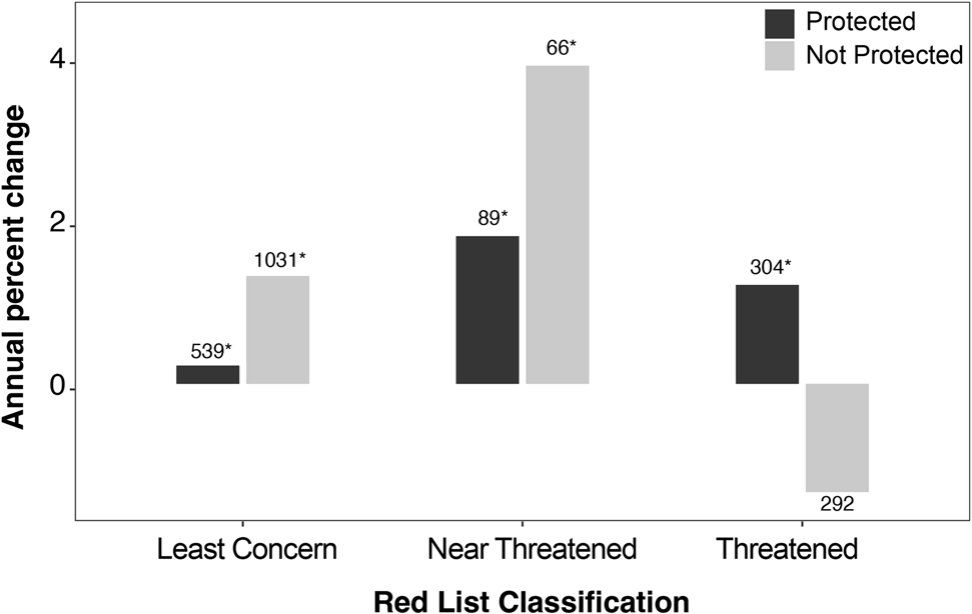
Median mammalian population percent change/year based on Red List Classification. The numbers above bars indicate the sample size of populations for each category. Asterisks indicate values that are significantly different from zero

Overall, mammalian populations both inside and outside terrestrial protected areas appear to be stable and perhaps slightly increasing with median percent changes of 0.71% (Wilcoxon test; *p* < 0.001) and 0.49% (Wilcoxon test; *p* < 0.001) respectively. Of the populations studied, 31.9% were Artiodactyls, an order of ungulates. Artiodactyl and carnivore populations were increasing inside and outside protected areas (Fig. 2). Within protected areas, Dasyuromorphia populations were decreasing, whereas proboscideans and perissodactyls were increasing. Chiroptera numbers were increasing outside of protected areas (Wilcoxon test; *p* < 0.001) (Fig. 2; Table 1). Populations classified as Threatened were increasing within protected areas (Wilcoxon test; *p* < 0.001), whereas those same populations did not show significant changes outside of protected areas (Wilcoxon test; *p* > 0.05) (Fig. 3). Near Threatened and Least Concern species on the IUCN Red List showed significant increases inside and outside protected areas (Wilcoxon test; *p* < 0.05) (Fig. 3).

**Table 1:** A comparison of mammal orders^a^

Order	Species in red list^b^	Species sampled in PAs	Species sampled outside PAs	Median percent change/year in PAs	Median percent change/year outside PAs
Afrosoricida	55	5	0	80.0	NA
Artiodactyla	336	83	80	0.28*	2.16*
Carnivora	297	55	43	0.83*	0.89*
Chiroptera	1332	15	42	− 5.66	2.84*
Cingulata	20	2	0	3.82	NA
Dasyuromorphia	72	7	1	− 19.2*	NA
Didelphimorphia	98	9	4	2.49	3.57
Diprotodontia	147	10	15	1.56	− 1.11
Erinaceomorpha	NA	1	0	NA	NA
Lagomorpha	96	5	8	4.02	− 3.00
Microbiotheria	1	1	1	13.4	− 5.95
Monotremata	5	1	0	NA	NA
Peramelemorphia	22	1	1	NA	NA
Perissodactyla	16	13	11	2.15*	− 4.35
Pholidota	8	1	0	NA	NA
Pilosa	10	2	0	− 10.7	NA
Primates	522	47	34	1.38	− 0.28
Proboscidea	3	3	3	2.22*	4.36
Rodentia	2375	91	83	− 2.78	− 0.77
Scandentia	23	3	0	–19.9	NA
Soricomorpha	NA	4	17	0	− 0.38
Total					

^a^Significant percent changes/year are asterisked. NA indicates fewer than 2 populations were sampled, so we did not analyze those groups, ^b^IUCN (2022)

### IUCN protected area categories

**Fig. 4:**
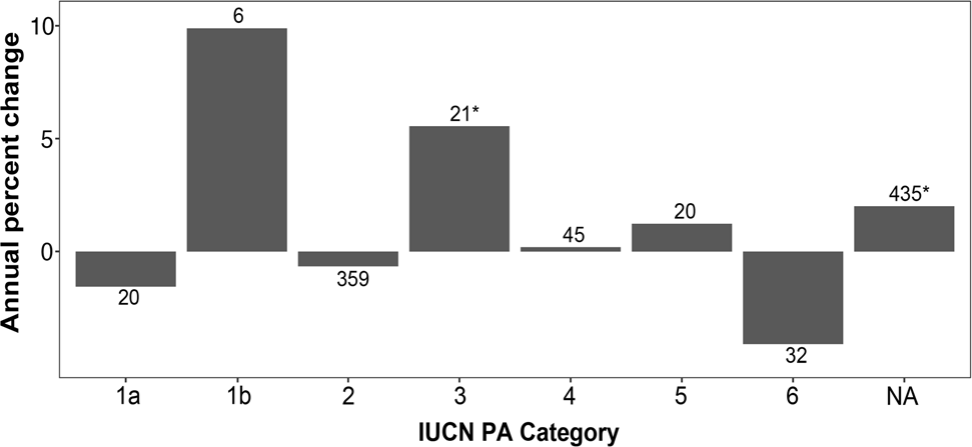
Median mammalian population percent change/year by protected area characteristics. The numbers above bars indicate the sample size of populations for each category. Asterisks indicate values that differ significantly from zero. The IUCN categories are: Ia-strict nature reserve, Ib-wilderness area, II-national park, III-natural monument or feature, IV-habitat/species management area, V-protected landscape, and VI-protected areas with sustainable use of natural resources

Within protected areas, annual percent change in mammal populations only increased significantly in category III, natural monuments (Wilcoxon test; *p* = 0.019) and for those areas that were not classified (NA) (Wilcoxon test; *p* < 0.001) (Fig. 4).

### Regions

**Fig. 5:**
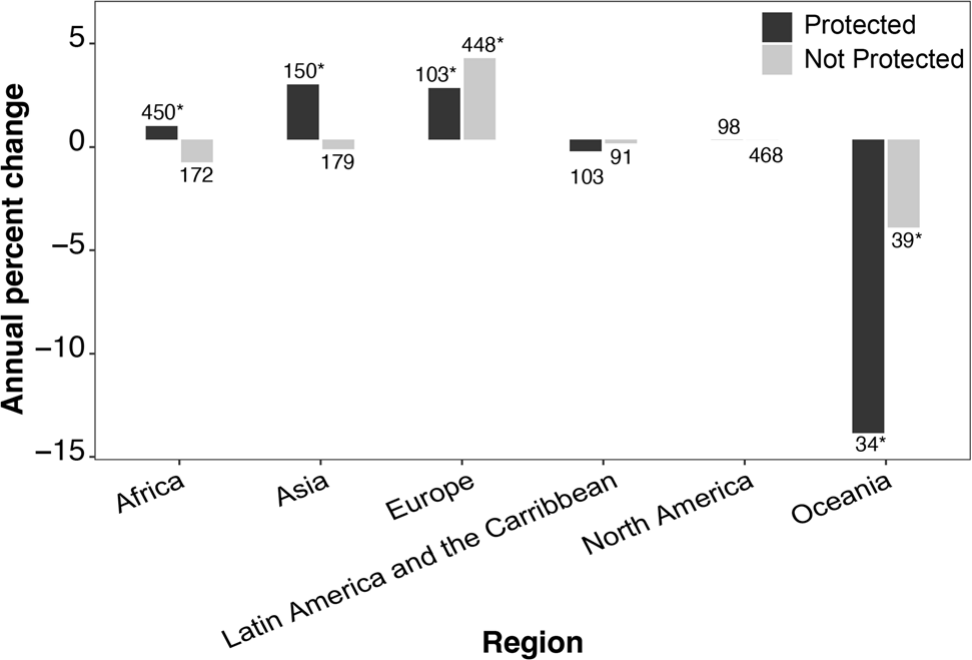
Median mammalian population percent change/year by region. The numbers above bars indicate the sample size of populations for each category. Asterisks indicate values that differ significantly from zero

In our analysis, 24.9% of the populations were located in Africa and all continents except Antarctica were represented (Table S1). Protected areas in Africa, Asia, and Europe showed a slight positive trend in mammalian population size (Wilcoxon test; *p* < 0.001), and unprotected populations in Europe were also increasing (Wilcoxon test; *p* < 0.001) (Fig. 5). Oceania had a significant decrease in both protected and unprotected areas (Wilcoxon test; *p* < 0.01) (Fig. 5).

### Biomes

**Fig. 6:**
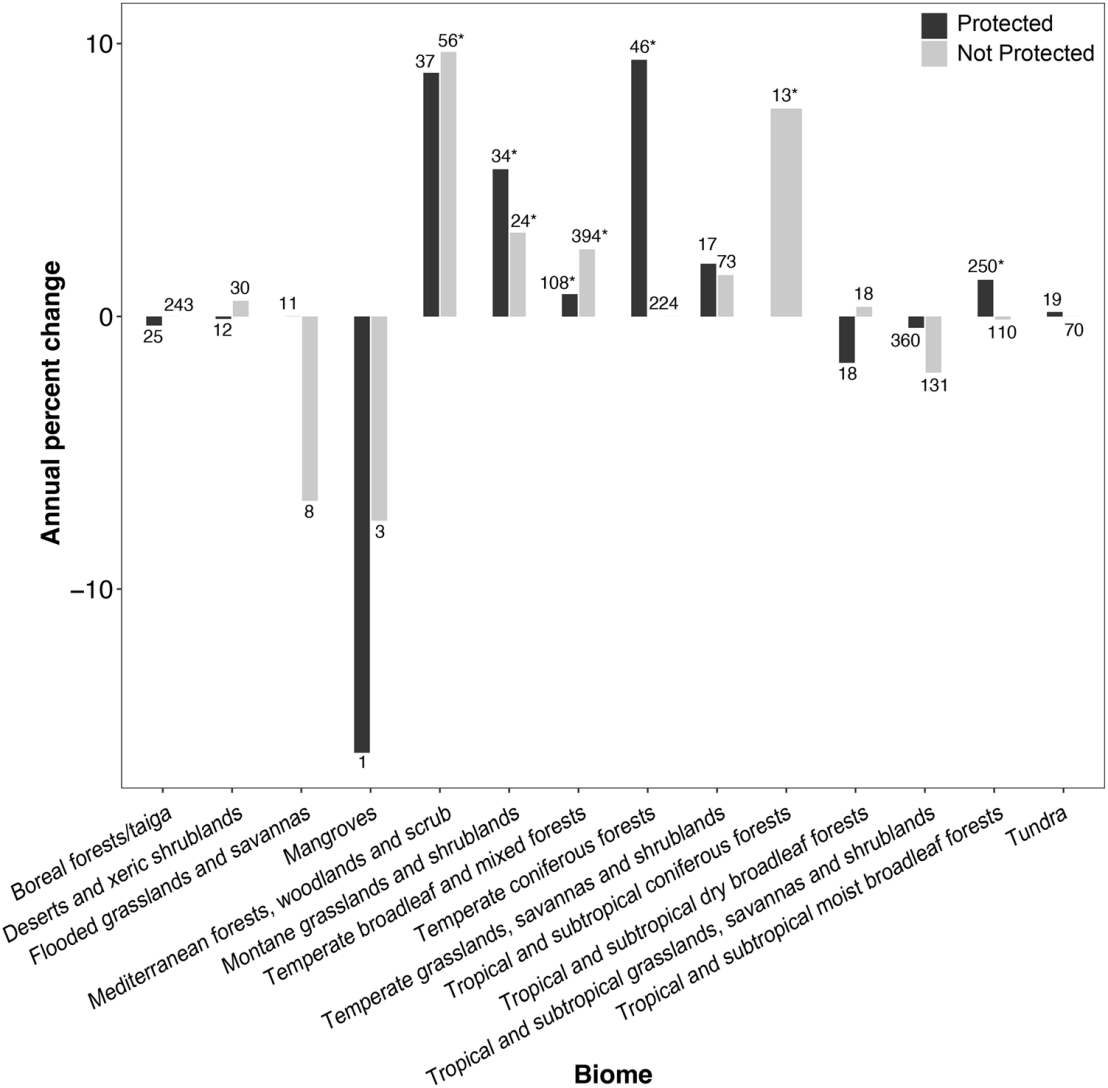
Median mammalian population percent change/year by region. The numbers above bars indicate the sample size of populations for each category. Asterisks indicate values that differ significantly from zero

All fourteen terrestrial biomes were represented (Table S1) with 20.0% of populations located in tropical and subtropical grasslands, savannas and shrublands. Protected areas were found in every biome except tropical and subtropical coniferous forests. Mammalian populations in montane grasslands and shrublands and temperate broadleaf and mixed forests showed increases in both protected and unprotected areas (Wilcoxon test; *p* < 0.05) (Fig. 6). Populations in protected areas had increases in tropical and subtropical moist broadleaf forests and temperate coniferous forests, whereas populations outside protected areas showed increases in Mediterranean forests, woodlands and scrub and tropical and subtropical coniferous forests (*p* < 0.05) (Fig. 6).

### Mixed effect model results

Mixed effects modeling indicated no significant relationship between annual percent change in mammal populations and PA area and remoteness. For the linear mixed effects model with protected area: area and remoteness as fixed effects and region as the random effect, area (*β* =  − 4.88, SE = 16.60) and remoteness (*β* =  − 1.15, SE = 9.00) had a negligible effect. The same was true when using order as a random effect: area (*β* =  − 3.78, SE = 16.60), remoteness (*β* =  − 3.15, SE = 8.84).

## Discussion

We found that protected areas can be important for mammal population recovery, but only in certain circumstances. Our results show Threatened mammals were doing better inside than outside of protected areas (Fig. 3). Protected areas are often designed and established to protect threatened species (Rodrigues et al. 2004, 2006) and our results show that they were effective overall at achieving this goal. Taylor et al. (2011) found that in Australia strictly protected areas (defined as IUCN categories I-IV) were successful at protecting threatened species when compared to other conservation measures. Since PAs are not often explicitly designed with species of Least Concern in mind, it is unsurprising that those species may be faring better outside of PAs. Even though many threatened species are not adequately protected by PAs (Williams et al. 2022), our findings indicate that threatened species in many PAs see population increases.

### IUCN protected area categories

Populations were not increasing to a greater degree across protected areas with higher levels of protection compared to those with lower levels of protection, but IUCN PA category III, also known as "natural monuments," was the only PA category where the median population trend significantly differed from zero with an increase of 5.56% (Fig. 4). Leroux et al. (2010) found that although the proposed ranking of PAs from most to least natural is Ia = Ib > II = III > IV = VI > V, categories III and Ib actually have the lowest Human Footprint. However, there are inconsistent management practices within the protection categories (Muñoz and Hausner 2013). Subsequently, Leberger et al. (2020) identified category III as having a high amount of forest cover and a low amount of forest loss, though these rates might be increasing. Thus, it seems that the low Human Footprint levels and intact forest in category III PAs may be beneficial to mammal populations.

### Regions

Previous work has shown that African mammals in protected areas were experiencing a decline (Western et al. 2009; Craigie et al. 2010), and our results indicate that in Africa, population change outside of protected areas does not significantly differ from zero and that populations may be slightly increasing within protected areas. There have been fewer longitudinal studies of Asian animals (de Silva 2016), but our results indicate that there was a slight positive trend among populations within protected areas. European mammals were increasing in all areas which reflects previous findings (Chapron et al. 2014; Carpio et al. 2021).

Oceania showed significant declines in mammalian populations both inside and outside of protected areas. This region is a biodiversity hotspot and has previously been identified as an area particularly vulnerable to extinctions (Kingsford et al. 2009; Jupiter et al. 2014). This may be, in part, because the region is overrepresented in mammal extinction risk research (Verde Arregoitia 2016). The region is also highly susceptible to habitat loss due to climate change, which can lead to mammal population declines (Taylor and kumar 2016; Baisero et al. 2020).

### Biomes

Although forested biomes are one of the biomes most susceptible to habitat loss and fragmentation (Hoekstra et al. 2005; Haddad et al. 2015), our results show that certain forested biomes had significant population recovery rates for mammals inside and outside of protected area. Globally, 13.5% of forested area fits in to one of the six IUCN protected area categories (Schmitt et al. 2009). Potapov et al. (2008) found that temperate broadleaf/mixed forests had the lowest proportion of intact forest landscape of any biome. Yet temperate forests have increased in area over the past 25 years (Keenan et al. 2015), and are the only biome in which total global habitat area is projected to increase by 2050 due to high rates of afforestation (Millennium Ecosystem Assessment 2005). It is unclear as to whether or not protected areas prevent deforestation from occurring inside them, and it may be variable by geographic region (Brandt et al. 2015; Brun et al. 2015; Cuenca et al. 2016). Regardless, our results suggest that when certain forested biomes are within a protected area, the mammal populations within them were able to increase. This reinforces previous results that protected areas are most effective when they maintain vegetation and curtail human land-use (Gray et al. 2016) as well as studies that have shown that intact forest is valuable for biodiversity conservation (Betts et al. 2017; Watson et al. 2018).

Our results do not show any significant relationship between percent change in mammal populations and area and remoteness of protected areas, which contradicts findings from previous studies (Barnes et al. 2016a, b; Schulze et al. 2018). It is possible that our remoteness proxy, travel time to cities (Weiss et al. 2018), is not capturing the same level of remoteness as other metrics such as: elevation, distance to major cities, slope, and distance to roads that have also been used (Joppa and Pfaff 2009). Our results indicate that larger protected areas are not necessarily better.

Although our results show some correlation between protected areas and mammal population increases, it can be difficult to make generalizations based on population data from protected areas because there is clear geographic and taxonomic bias (Geldmann et al. 2013). It is possible that populations within protected areas were already steady or increasing prior to the establishment of protected areas. Further research should attempt to account for confounding factors that may explain why populations inside protected areas appear to fare better, including habitat type, land use history, or other unidentified factors. Because protected areas are not randomly distributed across the globe this can also influence outcomes. Previous studies have utilized counterfactuals to account for this discrepancy when studying protected areas (Eklund et al. 2019; Geldmann et al. 2019; Black and Anthony 2022). Within conservation research, mammals are an overrepresented taxonomic group (Clark and May 2002). So, it is unclear if these trends will be reflected in other taxonomic groups. Additionally, highly cited journals have biases toward certain geographic areas such as temperate zones and wealthy countries (Collen et al. 2006; Martin et al. 2012), and there are fewer longitudinal population studies, which are getting more difficult to start and maintain (Clutton-Brock and Sheldon 2010; Schradin and Hayes 2017). Also, despite our efforts to identify populations that spanned protected and unprotected areas, as noted by Rodrigues and Cazalis (2020) there is spillover and connectivity between protected and unprotected areas. Our analysis is an effort to further fill in the gaps and provide more context to the status of mammal population trends and how they differ inside and outside of protected areas.

## Conclusion

Our results show that terrestrial protected areas can be important approaches for mammalian recovery and conservation, but success is taxon- and location-dependent. Protected areas are an important measure, but they alone cannot solve the ongoing biodiversity crisis. Overall, mammals classified as Threatened were increasing more within protected areas, whereas mammals classified as Least Concern or Near Threatened were increasing more outside protected areas. This indicates that PAs which contain Threatened species are important, and allocation of resources toward these areas should be prioritized. Some biomes and continents showed a significant percent increase/year which suggests that certain subsets of mammals in certain protected area environments may experience recovery over time. Thus, it is crucial to tailor recovery programs based on geographic location. Mammalian populations are generally increasing, but not substantially, so further work, such as more longitudinal studies of species and locations that have not yet been examined, is needed to reach global conservation objectives.

### Supplementary Information

Below is the link to the electronic supplementary material.

Supplementary file1 (XLSX 1449 KB)
